# Illness Perception and Quality of Life in Patients with Breast Cancer

**DOI:** 10.3390/cancers14051214

**Published:** 2022-02-25

**Authors:** Edyta Ośmiałowska, Jakub Staś, Mariusz Chabowski, Beata Jankowska-Polańska

**Affiliations:** 1 Division of Anesthesiologic and Surgical Nursing, Department of Nursing and Obstetrics, Faculty of Health Science, Wroclaw Medical University, 5 Bartla Street, 51-618 Wroclaw, Poland; edziaosmial@wp.pl; 2 Student Research Group No. 180, Faculty of Medicine, Wroclaw Medical University, 50-367 Wrocław, Poland; stas.jakub1@gmail.com; 3 Department of Surgery, 4th Military Teaching Hospital, 5 Weigla Street, 50-981 Wrocław, Poland; 4 Innovation and Research Center, 4th Military Teaching Hospital, 5 Weigla Street, 50-981 Wrocław, Poland; bianko@poczta.onet.pl

**Keywords:** breast cancer, quality of life, acceptance of illness, essence of disease, dispositional level of optimism, coping strategies

## Abstract

**Simple Summary:**

The declining average age of cancer patients may become a serious problem for health care systems and societies in general in the near future. For this reason, there is a need to fully understand the factors determining health-related quality of life in breast cancer patients, beyond clinical characteristics and sociodemographic factors. In our study, we aimed to demonstrate the relationship between illness perception and quality of life in breast cancer patients. The results of our study confirm the beneficial effect of positive illness perception on the intensity of symptoms related to cancer and treatment, as well as functional domains of EORTC QLQ-C30.

**Abstract:**

Introduction. In 2020, breast cancer was the most frequently diagnosed malignancy worldwide. The QoL level plays a role in assessing the effectiveness of the diagnosis and therapy and is a significant prognostic factor. The subject that is relatively less often addressed in the literature is the impact of psycho-social factors and health-related beliefs on QoL in breast cancer patients. The aim of the study was to assess the association of illness perception, the sense of coherence, and illness acceptance with QoL in breast cancer patients. Methods. The study included 202 women (mean age 53.0 ± 10.3) treated surgically for breast cancer at the Lower Silesian Oncology Centre. The following standardized questionnaires were used: Acceptance of Illness Scale (AIS), Mental Adjustment to Cancer (Mini-MAC), Quality of Life Questionnaires (EORTC QLQ-C30 and QLQ-BR23), The Multidimensional Essence of Disease and Illness Scale (MEDIS), and Life Orientation Test (LOT-R). Results. There is a statistically significant association between illness acceptance and QoL. There is a statistically significant association between the sense of coherence (life optimism—LOT-R) and QoL among breast cancer patients. There is a statistically significant association between illness perception and QoL. There was a statistically significant correlation between the increasing importance of illness as a dysfunction, decreasing QoL, and increasing intensity of symptoms and complaints. Conclusions. Patients with a high level of illness acceptance, with an optimistic disposition, and with a positive illness perception have better QoL within all the functional domains and experience lower intensity of cancer- and treatment-related symptoms as compared to those with low level of illness acceptance, with moderate optimism or a pessimistic disposition, and with neutral or negative illness perception.

## 1. Introduction

Breast cancer poses a growing challenge for healthcare systems all over the world. In 2020, it was the most frequently diagnosed malignancy worldwide, with new cases exceeding 2.61 million, i.e., 11.7% of all cancer cases reported that year. At the same time, it caused 685,000 deaths, which ranks it fifth in terms of cancer mortality. Incidence rates for breast cancer are particularly high in developed countries, whereas mortality rates are higher in developing countries [1]. Recent years have seen a statistically significant increase in breast cancer incidence in women under 50 years who lead an active professional and family life [2,3]. At the same time, as a result of screening programs and evermore effective treatment methods, the prognosis for patients diagnosed with malignant breast cancer has considerably improved, and their 5-year survival rate has increased [4,5,6]. However, due to low health awareness and treatment access barriers, breast cancer mortality remains high in developing countries [1,7,8].

Cancer affects numerous areas of life. Women diagnosed with breast cancer experience increased anxiety and are more susceptible to depressive disorders [9]. Long-term and burdensome treatment is associated with numerous adverse side effects, impairing daily functioning, changing the patient’s perception of their body, and altering their social roles [10]. Cancer diagnosis has an impact not only on the patient, but also on their family and friends, even after treatment has been completed [11]. 

Quality of life (QoL) assessment is of particular importance in the case of women with breast cancer [12]. The QoL level plays a role in assessing the effectiveness of the diagnostic and therapeutic process and is a significant prognostic factor [13,14]. QoL determinants in cancer patients can be divided into three groups: clinical, socio-demographic, and psycho-social. The impact of clinical variables (e.g., TNM stage, treatment used, symptom severity) and socio-demographic variables (e.g., age, education, marital status, place of residence) on QoL in patients treated for breast cancer has been extensively studied by various authors [15,16,17]. The subject that is relatively less often addressed in the literature is the impact of psycho-social factors and health-related beliefs on QoL in breast cancer patients. A better understanding of factors that negatively affect QoL may form the basis for the modification and improved personalization of the existing breast cancer treatment protocols. 

The aim of the study was to assess the association of illness perception, the sense of coherence, and illness acceptance with QoL in breast cancer patients.

## 2. Material and Methods

The study included a group of 202 women treated for breast cancer at the Lower Silesian Oncology Centre. The patients were enrolled based on the following inclusion criteria: age between 18 and 75 years; diagnosis of early non-invasive breast cancer; surgical intervention used at any treatment stage; and voluntary consent to participate in the study. The exclusion criteria were as follows: treatment without surgical intervention; coexistence of any other cancer or severe chronic disease, which might affect the patient’s perception of their health status; and severe depression requiring specialist treatment. The study protocol was approved by the Medical University Bioethics Committee (approval no. KB-196/2018). The study was conducted among patients reporting for follow-up appointments at the Oncology Clinic and the Surgical Oncology Clinic at least three months after the surgical procedure. Before the commencement of the study, all the participants were informed about the purpose of the study, full anonymity, and the possibility to withdraw from the study at any stage. Having completed the consent form, the patients received a set of questionnaires for self-completion in the presence of a researcher.

Quality of life was assessed with the European Organization for Research and Treatment of Cancer Quality of Life Questionnaire Core 30 (EORTC QLQ-C30). QLQ-C30 is a standardized tool commonly used in clinical studies [18]. The questionnaire is composed of 30 items assessing the patient’s global health status/QoL, five functional domains (physical, role, cognitive, emotional, and social functioning), and the intensity of individual symptoms (fatigue, nausea and vomiting, pain, dyspnoea, sleep disturbance, appetite loss, constipation, diarrhea, and financial difficulties caused by the disease). In the study, we also used the European Organization for Research and Treatment of Cancer Quality of Life Questionnaire for Breast Cancer 23 (EORTC QLQ-BR23), which is a module assessing QoL in breast cancer patients. It consists of five cancer-specific scales (body image and sexual functioning, and three symptom scales: systemic therapy side effects, breast symptoms, and arm symptoms). Furthermore, the instrument includes questions pertaining to sexual enjoyment, future perspective, and being upset by hair loss. The scores in all the EORTC QLQ-C30 and QLQ-BR23 scales were converted to range from 0–100, so that the highest score denoted the highest intensity of the variable studied. The Polish versions of the EORTC QLQ-C30 and QLQ-BR23 had been validated as reliable instruments for assessing QoL in breast cancer patients [19]. 

Illness acceptance was measured with the Acceptance of Illness Scale (AIS), comprising eight items pertaining to the limitations, problems with adaptation, the lack of self-efficacy, and lowered self-esteem experienced by patients suffering from a chronic disease. Each of the eight items is assigned an acceptance score on a 5-point scale, where 1 denotes “strongly agree” and 5 “strongly disagree”. The total score can range from 8 to 40, with higher scores indicating a higher level of illness acceptance. There are three levels of illness acceptance depending on the score range: low—8–18 pts.; moderate—19–29 pts.; and high—30–40 pts. In the study, we used the Polish adaptation of the AIS developed by Juczyński. The reliability of the Polish version of the AIS is close to the original in terms of accuracy and consistency (Cronbach’s α = 0.82) [20,21].

The Polish adaptation of the Revised Life Orientation Test (LOT-R) by Poprawa and Juczyński comprises ten items, six of which diagnose the level of dispositional optimism. The total score is the sum of the six item scores. Items 1, 4, and 10 are positive statements, whereas 3, 7, 9 are negative. The respondent rates a given statement on a 5-point Likert scale, where 0 denotes “strongly agree” and 4 “strongly disagree”. Before summing up the scores from statements 3, 7, and 9, the scoring is reverse-coded in the following way: 0 = 4, 1 = 3, 2 = 2, 3 = 1, 4 = 0. The total score ranges from 0 to 24. The higher the score, the higher the level of optimism [22].

The Mental Adjustment to Cancer (Mini-MAC) is used to assess coping strategies in cancer patients. The scale is composed of 29 statements and measures four strategies of coping with cancer—two constructive ones (fighting spirit and positive redefinition) and two negative ones (anxious preoccupation and helplessness–hopelessness) [23].

The Multidimensional Essence of Disease and Illness Scale (MEDIS) designed by Sak and Sagan contains statements describing various meanings of “being ill”. According to the authors, the instrument examines general beliefs pertaining to the situation of being ill based on colloquial expressions. The scale comprises 28 items assigned to five descriptive factors: self-realization constraints (SC) (10 items); mental dysfunction (MD) (5 items); physical dysfunction (PD) (5 items); infection (IN) (4 items); and social withdrawal (SW) (4 items). The items are rated on a 5-point Likert scale, with 1 standing for “strongly disagree” and 5 “strongly agree”. The instrument allows for assessing the extent to which the respondent agrees with a given description of being ill. The higher the mean score in a given dimension (min—1.0; max—5.0), the greater the importance assigned to a given dimension describing the essence of being ill [24].

The patients’ clinical and socio-demographic data were obtained from their medical records and based on the authors’ own questionnaire. 

Qualitative variables (measured on nominal and ordinal scales) were presented in contingency tables as numbers (*n*) and percentages (%). The strength of an association between two variables was measured with the chi-squared test. The Kolmogorov–Smirnov test or the Shapiro–Wilk test, depending on the sample size, was used to test whether the distribution of the quantitative variables conformed to a normal distribution. Means (M), standard deviations (SD), medians (Me), lower and upper quartile values (Q1 and Q3), and the variation range (Min and Max) were calculated for all the quantitative variables. One-way analysis of variance (ANOVA) was used to compare the mean values of quantitative variables in several groups. Before that, it was verified whether a given variable in each of the groups studied had a normal distribution (Shapiro–Wilk test or Kolmogorov–Smirnov test) and equal variances (Bartlett’s test and Levene’s test). If the probability corresponding with the value of the F-distribution was lower than the significance level adopted (*p* < 0.05), it was verified which group’s mean significantly differed from the others. For that purpose, multiple comparison tests were conducted (post hoc). We used Tukey’s HSD (honestly significant difference) test. The analyses were performed using STATISTICA v. 12.5 software and Excel spreadsheet.

## 3. Results

### 3.1. General Clinical and Socio-Demographic Characteristics of the Patients Studied

The study included 202 patients aged 26–75 years (mean age 53.0 ± 10.3), diagnosed with breast cancer and treated surgically. The patients were examined during follow-up appointments at an oncology clinic or a surgical oncology clinic. The vast majority of the women had higher (39.6%) and secondary (38.6%) education, and most of them were professionally active (63.9%). The majority of the patients lived in cities (77.2%) and perceived their financial standing as good (63.4%). Among the women studied, 75.7% were in a stable relationship, and 85.1% had children. The results are presented in Table 1. 

In terms of disease duration, the largest group of respondents were women who suffered from breast cancer from 1 to 2 years (52.4%). In 22.8% of the patients, disease duration was 2–5 years, and in 24.8%, over 5 years. Over half of the respondents were diagnosed with cancer after they detected the tumor themselves during self-examination through palpation (53%). In 25.7% of the women, breast cancer was diagnosed following a screening mammogram, and in 21.3%, during a doctor’s visit (21.3%). The most commonly diagnosed comorbidities in the patients studied were hypertension (28.7%), diabetes (14.4%), and rheumatoid arthritis (5.9%). No other disease, apart from breast cancer, was diagnosed in 60.4% of the women. Over 91.6% of the patients undergoing breast cancer treatment were women who had received a cancer diagnosis for the first time. The most frequently reported complaints associated with the disease were low mood (57.9%), pain (47.0%), hair loss (37.1%), and arm swelling after axillary lymphadenectomy (30.7%). The following clinical manifestations of the disease according to the TNM classification were most prevalent in the patients studied: tumor size T2 (50%) and T1 (38.6%); and lymph node involvement N1 (35.6%) and N2 (4.5%). In 57.9% of the respondents, no lymph node metastasis was observed (N0). As for the presence of distant metastasis (M): M1 designation was reported in 1.5% of the patients; Mx in 24.8%; and M0 in 73.3%. All the women underwent surgical intervention. Additionally, 69.3% were treated with chemotherapy, 55.9% with radiotherapy, and 39.1% with hormone therapy. The study group included patients who were administered more than one type of treatment (Table 2).

### 3.2. Comparative Analyses of QoL Depending on Selected Psycho-Social Variables

- Analysis of QoL assessed with the QLQ-C30 relative to the level of illness acceptance assessed with the AIS in the breast cancer patients studied.

The comparative analysis of QoL assessed with the EORTC QLQ-C30 relative to the level of illness acceptance assessed with the AIS revealed statistically significant differences with respect to all the QoL domains of the QLQ-C30 questionnaire. Women with a high level of illness acceptance had higher QoL in all the functional domains (Table 3). 

A similar relationship was observed with respect to symptom intensity. Namely, women with a higher level of illness acceptance experienced a lower intensity of breast cancer symptoms. There were no statistical differences regarding the sleep disturbance domain (SL). In all the women studied, the most troublesome and QoL-lowering symptoms were fatigue (FA), sleep disturbance (SL), and financial difficulties (FI). The group with a low level of illness acceptance presented a high level of pain (PA). An additional problem observed in this group was appetite loss (AP). The results are presented in Table 4.

b. Analysis of QoL assessed with the EORTC QLQ-BR23 module relative to the level of illness acceptance assessed with the AIS in the breast cancer patients studied.

The comparative analysis of QoL assessed with the QLQ-BR23 questionnaire in patient groups differing with respect to the level of illness acceptance revealed better functioning and higher QoL in the body image domain (BRBI) among patients with a higher level of illness acceptance. A similar relationship was observed for the sexual functioning domain (BRSEF) and the sexual enjoyment domain (BRSEE). The exception was the future perspective domain (BRFU), with the highest scores observed in the group with a moderate level of illness acceptance and the lowest in the group with a high level of illness acceptance. The comparative analysis pertaining to cancer-related symptom scales revealed a higher level of symptom intensity and a greater negative impact on daily functioning among patients with a low level of illness acceptance. These values were the lowest among patients with a high level of illness acceptance. The exception was the “upset by hair loss” domain, with no statistically significant differences observed between the patient groups. The results are presented in Table 5.

c. Analysis of QoL assessed with the EORTC QLQ-C30 relative to the level of coherence in the breast cancer patients studied.

The comparative analysis of QoL measured with individual functional domains in patient groups differing with respect to the level of optimism revealed statistically significant differences between these groups. As for the global health status/QoL (QL), the higher the optimism level, the higher the quality of life (QoL). In terms of physical (PF), role (RF), and social (SF) functioning, the highest scores were obtained by the group displaying moderate optimism, followed by the group with an optimistic disposition, and finally, that with a pessimistic disposition. With regard to the cognitive (CF) and emotional (EF) functioning domains, the highest QoL scores were observed in the group with an optimistic disposition, and the lowest in the group with a pessimistic disposition. The results are presented in Table 6.

The comparative analysis of QoL related to cancer symptoms revealed statistically significant, higher symptom intensity in all the symptom scales apart from fatigue (FA) and sleep disturbance (SL) in the group of women with a pessimistic disposition or moderate optimism. The lowest symptom intensity was observed in the group displaying an optimistic disposition. The results are presented in Table 7.

d. Analysis of QoL assessed with the QLQ-BR23 module relative to the level of coherence in the breast cancer patients studied.

The comparative analysis of QoL measured with the BR23 module revealed higher QoL scores in the functional domains among women with an optimistic disposition.

As for the BR23 symptom scales, a statistically significant difference and lower intensity of cancer symptoms in patients with an optimistic disposition was observed only in the systemic therapy side effects domain (BRST). In terms of the other domains, there were no differences with respect to the level of coherence among the respondents. All the patients reported that they were most upset by hair loss (BRHL). The results are presented in Table 8.

e. Analysis of QoL assessed with the EORTC QLQ-C30 in individual functional domains relative to illness perception in the breast cancer patients studied.

In the group of patients with a negative illness perception (MEDIS > 95 pts.), the global health status/QoL score (QL) was significantly lower than in those patient groups who had a positive or neutral perception of their illness. The physical, role, emotional, cognitive, and social functioning scores were also lower among the women who had a negative illness perception as compared with those with a neutral or positive perception. The results are presented in Table 9. 

The assessment of cancer-related symptom intensity measured with the EORTC QLQ-C30 questionnaire relative to illness perception measured with the MEDIS questionnaire demonstrated statistically significant, higher symptom intensity in all the domains among women who had a negative perception of their illness. There was a relationship between illness perception and the intensity of all the symptoms measured—the more negative the perception, the greater the intensity of breast cancer symptoms. The results are presented in Table 10.

f. Analysis of QoL assessed with the EORTC QLQ-BR23 module relative to illness perception in the breast cancer patients studied.

The analysis of QoL assessed with the BR23 module in individual functional domains revealed statistically significant differences in the QoL level and cancer- and treatment-related symptom intensity relative to illness perception. Women with a positive illness perception obtained higher scores in all the QoL domains except for sexual enjoyment (BRSEE), with no statistically significant differences observed in that particular domain. On the other hand, a more negative illness perception translated into greater cancer-related symptom intensity in all the domains of the BR23 questionnaire (Table 11). 

We found a statistically significant correlation between the majority of the MEDIS domains and the QoL assessment and symptom intensity. An increase in the importance of self-realization constraints was associated with a decrease in QoL in all the functional domains and greater intensity of cancer-related symptoms. The exception was the sleep disturbance domain (SL), with no differences observed in relation to illness perception. 

Furthermore, an increase in the importance of mental dysfunction (DP) was associated with a decrease in QoL in all the functional domains and greater intensity of cancer-related symptoms. The exception was the sexual enjoyment domain (BRSEE), with no differences observed in relation to illness perception.

An increase in the importance of physical dysfunction (DF) was associated with a decrease in QoL in all the functional domains, except for emotional (EF) and cognitive (CF) functioning, and greater intensity of cancer-related symptoms in all domains, except for dyspnoea (DY), appetite loss (AP), financial difficulties (FI), and sexual enjoyment (BRSEE). An increase in the importance of infection (IN) was associated with a decrease in QoL in all the functional domains and greater intensity of cancer-related symptoms in all the domains, except for dyspnoea (DY), sleep disturbance (SL), constipation (CO), financial difficulties (FI), sexual functioning (BRSEF), and sexual enjoyment (BRSEE). An increase in the importance of social withdrawal (WS) was associated with a decrease in QoL in all the functional domains and greater intensity of breast cancer symptoms in all domains (Table 12).

### 3.3. Comparative Analyses of the Illness Acceptance Level and Coherence Depending on the Type of Coping Strategies Used

The next stage of the study involved comparative analyses between the type of the strategies used to cope with cancer, the degree of their use, and the level of illness acceptance (AIS), and optimism (LOT-R). The results showed that the level of dispositional optimism increased along with the degree of using constructive coping strategies. The patient group that displayed a low degree of using constructive strategies predominantly included women with a pessimistic disposition (80%). In contrast, the group with a high degree of using such strategies mostly included women presenting an optimistic disposition (51.2%) and moderate optimism (36.2%).

A similar relationship was observed for the illness acceptance level—the higher the degree of using constructive strategies, the higher the level of illness acceptance. The results are presented in Table 13.

In the group using destructive strategies, the trend was the opposite—patients with a low degree of using destructive strategies presented a higher level of dispositional optimism (sten scores). The patient group displaying a low degree of using destructive strategies predominantly included women with an optimistic disposition (60.8%), and the group with a high degree of using destructive strategies mostly included women presenting moderate optimism (64.7%) and a pessimistic disposition (35.3%) (Table 14). 

A similar relationship was observed for illness acceptance. Namely, the higher the level of illness acceptance, the lower the degree of using destructive strategies to cope with cancer.

## 4. Discussion

In light of current epidemiological trends, i.e., an increase in breast cancer morbidity, especially among women aged <50 years, this type of cancer poses an important public health problem [1,2]. Cancer mortality constitutes a significant burden for healthcare systems worldwide [25,26]. Extensive screening programs and advancements in therapy personalisation and new treatment methods offer hope for a considerable death rate reduction in the future [27,28]. Therefore, the assessment of the quality of life in breast cancer patients should be an essential element of healthcare, oriented towards improving the comfort and functioning of these patients during and after cancer treatment.

All the respondents in the study underwent the surgical treatment. To avoid the bias associated with early surgical factors, i.e., post-operative pain, surgical site infection, etc., the study authors maintained a three-month interval from the procedure before conducting a questionnaire assessment.

The present study demonstrated a significant reduction in QoL among the breast cancer patients analysed. Our findings point to a negative impact of cancer on all the functional scales studied. When compared with reference values for the general population in Poland, the greatest differences were observed in the role and physical functioning domains [29]. The analysis of QoL scores for the symptom scales demonstrated the highest symptom intensity for sleep disturbance and fatigue. As for breast cancer-specific symptoms, the most troublesome one was being upset by hair loss. The greatest difference relative to the reference values was observed for financial difficulties [27]. The results of the present study related to the global health status/QoL and symptom scales are similar to those obtained in other centres, except for the clearly higher intensity of financial difficulties among breast cancer patients in Malaysia [30]. On the other hand, our results were significantly different in terms of the QLQ-C30 functional scales and QLQ-BR23 functional and symptom scales as compared to the findings from other studies. In our study, the patients had good future perspective but, at the same time, low body image and sexual functioning scores [30,31,32]. These differences are concerning and give reason to consider adequate interventions in the future. 

Despite the fact that most of the respondents assess their financial situation as good and very good in the authors’ own questionnaire, in terms of the QLQ-C30 symptom scales, the greatest difference relative to the reference values was observed in the financial difficulties domain. The mean result in the study group was 30.0 ± 30.0, whereas the reference value for the Polish population is 15.5 ± 24.9 [29]. Individual cancer-associated expenses may constitute a considerable economic burden for patients [33,34]. A study by Andritch et al. showed that financial problems were one of the factors negatively affecting patients’ QoL [35]. Our study demonstrated a significant correlation between the increase in the importance of self-realisation constraints, mental dysfunction and social withdrawal (MEDIS), and the intensity of financial difficulties (QLQ-C30). Financial security is an important factor affecting the functioning of breast cancer patients. Therefore, this issue requires additional, in-depth analysis. 

Illness acceptance in the group studied (28.5 ± 8.5) was at a moderate, nearly high level. The comparative analysis revealed a statistically significant correlation between the participants’ reported QoL and their illness acceptance level. The patients with a higher level of illness acceptance displayed higher QoL and lower intensity of all cancer- and treatment-related symptoms. These results corroborate the findings obtained in other studies on breast cancer patients [36,37]. Such a relationship was also observed in studies conducted among groups of patients suffering from other cancers and chronic diseases [38,39,40]. It is worth emphasising the high intensity of pain observed in the patient group with a low level of illness acceptance. A similar association was found among women, differing with respect to illness perception (MEDIS). The current state of knowledge about pain perception and factors responsible for its modulation points to an important role of the emotional sphere in the nociceptive pain experience [41]. Pain is a factor significantly decreasing QoL in breast cancer patients [42]. Further research is needed to explore this association. In their study, Kozieł et al. demonstrated that the level of illness acceptance in a group of breast cancer patients was lower if the patient experienced increased anxiety or depression [37]. The destructive impact of anxiety on the level of illness acceptance was also confirmed in a study by Dryhinicz et al. [43]. Due to the prevalence of anxiety among cancer patients, this association may play an important role when planning activities oriented towards enhancing illness acceptance [44]. According to Jankowska-Polańska et al., a higher level of illness acceptance has a positive effect on therapy adherence in patients with hypertension. This beneficial effect was observed for both pharmacological and non-pharmacological treatment [45]. Illness acceptance allows for a rational assessment of one’s situation and undertaking health maintenance activities, which may increase QoL [46,47]. 

The analysis of the life orientation test results showed that an optimistic disposition was predominant in the group studied. The comparative analysis in our study showed higher QoL and lower symptom intensity in patients with an optimistic disposition. Our results are in line with the findings published by Hiensch et al. and Rohani et al., who demonstrated a positive correlation between the sense of coherence and health-related quality of life [48,49].

According to Motyka et al., a strong sense of coherence can shorten the time between noticing the symptoms and having a doctor’s appointment. It is likely that individuals with a strong sense of coherence create a more adequate image of their health, which is conducive to shaping sought-after health behaviors [50]. In their study, Kaźmierczak et al. demonstrated a strong relationship between the acceptance of illness and the sense of coherence [51]. The results of our study show that both a high level of illness acceptance and a more optimistic disposition have an effect on choosing a constructive strategy of coping with cancer. The choice between a constructive and destructive model may significantly affect QoL in breast cancer patients [52,53]. 

An important aspect of the present study involved determining the association between illness perception and QoL in the patients analyzed. The study results showed that women with breast cancer form a very diverse group in terms of illness perception. The comparative analysis demonstrated that the patients with a negative illness perception had poorer QoL as compared to those who perceived their illness positively or neutrally. The very concept of illness perception is based on the assumption that the patient’s response to the disease is shaped by their personal experiences. A study by Lee et al. showed a significant positive relationship between illness perception and the sense of well-being among breast cancer patients [54]. Vollmann et al. assessed the impact of negative illness perception at the beginning of treatment in patients with non-small-cell lung cancer. A more negative illness perception at the beginning of treatment was associated with poorer functioning and lower QoL both at the beginning and at the end of treatment [55]. 

The limitations of this publication result from the limited literature available on the subject and the difficulties in eliminating the influence of clinical and sociodemographic factors affecting the quality of life of patients with breast cancer. In order to reduce the possible bias of the results, the study group was qualified based on strict inclusion criteria.

Further research on the role of illness perception among women with breast cancer may provide important information on how to plan care, education, and counselling for this group of patients.

## 5. Conclusions

1. There is a statistically significant association between illness acceptance and QoL. Patients with a high level of illness acceptance have higher QoL within all functional domains and experience lower intensity of cancer- and treatment-related symptoms.
2. There is a statistically significant association between the sense of coherence (life optimism—LOT-R) and QoL among breast cancer patients. Patients with an optimistic disposition have higher QoL and lower symptom intensity in all the EORTC QoL-C30 and BR23 domains as compared to patients displaying moderate optimism or a pessimistic disposition.
3. There is a statistically significant association between illness perception and QoL. Patients with a positive illness perception have higher QoL in all the functional domains and lower intensity of cancer- and treatment-related symptoms as compared to those who perceive their illness neutrally or negatively. There was a statistically significant correlation between the increasing importance of illness as a dysfunction, decreasing QoL, and increasing intensity of symptoms and complaints.

## Figures and Tables

**Table 1:** General socio-demographic characteristics of the patients studied.

Variable	*n* (%)
1. Age (years):	
*M* ± *SD*	53.0 ± 10.3
*Me* [*Q*_1_; *Q*_3_]	52 [45; 61]
*Min*–*Max*	26–75
2. Education:	
Primary	9 (4.5%)
Vocational	35 (17.3%)
Secondary	78 (38.6%)
Higher	80 (39.6%)
3. How would you assess your financial standing?	
Very good	36 (17.8%)
Good	128 (63.4%)
Insufficient	35 (17.3%)
Poor	3 (1.5%)
4. Are you professionally active?	
Yes	129 (63.9%)
No	73 (36.1%)
5. Residence:	
City	156 (77.2%)
Village	46 (22.8%)
6. Are you in a stable relationship?	
Yes	153 (75.7%)
No	49 (24.3%)
7. Do you have children?	
Yes	163 (80.7%)
No	39 (19.3%)
7a. No. of children:	
0	39 (19.3%)
1	72 (35.6%)
2	76 (37.6%)
3 or more	15 (7.4%)
7b. Age of the youngest child:	
*M* ± *SD*	22.6 ± 11.7
*Me* [*Q*_1_; *Q*_3_]	23 [13; 31]
*Min*–*Max*	1–52
7b. Age of the eldest child:	
*M* ± *SD*	27.6 ± 11.7
*Me* [*Q*_1_; *Q*_3_]	27 [16; 38]
*Min*–*Max*	1–54

*M*—mean; *SD*—standard deviation; *Me*—median; *Q*_1_—lower quartile; *Q*_3_—upper quartile; *Min*—lowest value; *Max*—highest value; *n*—number; %—percentage.

**Table 2:** Clinical characteristics of the patients treated for breast cancer.

Variable	*n* (%)
How long have you had breast cancer?	
1–2 years	106 (52.4%)
2–5 years	46 (22.8%)
Over 5 years	50 (24.8%)
How were you diagnosed with breast cancer?	
I detected it myself	107 (53.0%)
During a doctor’s visit	43 (21.3%)
After a screening mammogram	52 (25.7%)
Do you have any of the following chronic diseases?	
Diabetes	29 (14.4%)
Hypertension	58 (28.7%)
Renal failure	5 (2.5%)
Rheumatoid arthritis	12 (5.9%)
Asthma/COPD	4 (2.0%)
No	122 (60.4%)
Were you diagnosed with metastases? If so, to what organs?	
No	107 (53.0%)
Yes, to the lymph nodes	64 (31.7%)
Yes, to the bones	4 (2.0%)
Yes, to the brain	1 (0.5%)
Yes, to the liver	4 (2.0%)
Yes, to other organs	22 (9.2%)
Which of the following types of treatment did you undergo?	
Surgery	202 (100%)
Chemotherapy	140 (69.3%)
Radiotherapy	113 (55.9%)
Hormone therapy	79 (39.1%)
Which of the following cancer-related complaints do you find most troublesome?	
Pain	95 (47.0%)
Arm swelling after axillary lymphadenectomy	62 (30.7%)
Hair loss	75 (37.1%)
Low mood	117 (57.9%)
Other (restlessness, fatigue, nausea and vomiting)	10 (5.0%)
Tumour size (T):	
Tx	4 (2.0%)
T1	78 (38.6%)
T2	102 (50.5%)
T3	16 (7.9%)
T4	2 (1.0%)
Lymph node involvement (N):	
N0	117 (57.9%)
N1	72 (35.6%)
N2	9 (4.5%)
N3	1 (0.5%)
Nx	3 (1.5%)
Distant metastasis (M):	
M0	148 (73.3%)
M1	3 (1.5%)
M2	1 (0.5%)
Mx	50 (24.8%)

%—percentage.

**Table 3:** The results of QoL assessment (EORTC QLQ-C30 functional scales) in patient groups differing with respect to the level of illness acceptance; and the results of the analysis of variance.

QoL Assessed with EORTC QLQ-C30	Level of Illness Acceptance (AIS)			ANOVA *p*
	High Acceptance 30–40 pts.	Moderate Acceptance 19–29 pts.	Low Acceptance 8–18 pts.	
	*n* = 101	*n* = 69	*n* = 32	
Global health status/QoL (QL)				**0.001**
*M* ± *SD*	64.7 ± 16.3	57.7 ± 18.6	37.8 ± 24.4	
*Me* (*Q*_1_; *Q*_3_)	67 [50; 75]	58 [50; 67]	33 [17; 58]	
*Min*–*Max*	17–100	8–100	0–83	
Physical functioning (PF)				**0.001**
*M* ± *SD*	40.4 ± 24.4	22.5 ± 13.9	19.9 ± 13.7	
*Me* (*Q*_1_; *Q*_3_)	43 [18; 60]	20 [13; 27]	20 [7; 27]	
*Min*–*Max*	7–87	0–73	0–60	
Role functioning (RF)				**0.001**
*M* ± *SD*	39.1 ± 28.3	18.1 ± 18.9	15.0 ± 17.1	
*Me* (*Q*_1_; *Q*_3_)	33 [17; 67]	17 [0; 17]	17 [0; 17]	
*Min*–*Max*	0–100	0–67	0–67	
Emotional functioning (EF)				**0.001**
*M* ± *SD*	53.1 ± 25.3	41.5 ± 26.0	30.1 ± 19.9	
*Me* (*Q*_1_; *Q*_3_)	58 [33; 75]	33 [17; 67]	25 [17; 33]	
*Min*–*Max*	0–92	0–100	0–92	
Cognitive functioning (CF)				**0.001**
*M* ± *SD*	43.2 ± 32.5	25.1 ± 23.5	18.6 ± 20.3	
*Me* (*Q*_1_; *Q*_3_)	33 [17; 67]	17 [0; 33]	17 [0; 33]	
*Min*–*Max*	0–100	0–100	0–83	
Social functioning (SF)				**0.001**
*M* ± *SD*	47.4 ± 29.1	31.2 ± 22.7	22.3 ± 20.2	
*Me* (*Q*_1_; *Q*_3_)	50 [29; 67]	33 [17; 50]	17 [0; 33]	
*Min*–*Max*	0–100	0–100	0–100	

*M*—mean; *SD*—standard deviation; *Me*—median; *Q*_1_—lower quartile; *Q*_3_—upper quartile; *Min*—lowest value; *Max*—highest value; *n*—number; values in bold indicate statistical significance (*p* < 0.05).

**Table 4:** The results of QoL assessment (EORTC QLQ-C30 symptom scales) in patient groups differing with respect to the level of illness acceptance and the results of the analysis of variance.

QLQ-C30 Symptom Scales	Level of Illness Acceptance (AIS)			ANOVA *p*
	Low Acceptance 8–18 pts.	Moderate Acceptance 19–29 pts.	High Acceptance 30–40 pts.	
	*n* = 32	*n* = 69	*n* = 101	
Fatigue (FA)				**<0.001**
*M* ± *SD*	51.0 ± 21.5	42.7 ± 21.4	34.3 ± 21.0	
*Me* (*Q*_1_; *Q*_3_)	44 [33; 67]	33 [33; 56]	33 [22; 44]	
*Min*–*Max*	11–100	0–100	0–100	
Nausea and vomiting (NV)				**<0.001**
*M* ± *SD*	35.9 ± 36.4	17.1 ± 24.6	6.4 ± 17.8	
*Me* (*Q*_1_; *Q*_3_)	33 [0; 67]	0 [0; 33]	0 [0; 0]	
*Min*–*Max*	0–100	0–100	0–100	
Pain (PA)				**<0.001**
*M* ± *SD*	44.8 ± 24.5	31.2 ± 24.9	21.1 ± 23.0	
*Me* (*Q*_1_; *Q*_3_)	50 [33; 67]	33 [17; 50]	17 [0; 33]	
*Min*–*Max*	0–100	0–100	0–100	
Dyspnoea (DY)				**<0.001**
*M* ± *SD*	34.4 ± 35.4	10.8 ± 23.4	10.6 ± 20.0	
*Me* (*Q*_1_; *Q*_3_)	33 [0; 67]	0 [0; 0]	0 [0; 0]	
*Min*–*Max*	0–100	0–100	0–67	
Sleep disturbance (SL)				0.278
*M* ± *SD*	46.9 ± 31.5	42.6 ± 33.0	37.0 ± 34.3	
*Me* (*Q*_1_; *Q*_3_)	67 [33; 67]	33 [33; 67]	33 [0; 67]	
*Min*–*Max*	0–100	0–100	0–100	
Appetite loss (AP)				**<0.001**
*M* ± *SD*	40.6 ± 31.4	24.2 ± 28.5	12.2 ± 22.5	
*Me* (*Q*_1_; *Q*_3_)	33 [0; 67]	33 [0; 33]	0 [0; 33]	
*Min*–*Max*	0–100	0–100	0–100	
Constipation (CO)				**0.001**
*M* ± *SD*	31.2 ± 31.6	24.6 ± 19.5	14.9 ± 24.3	
*Me* (*Q*_1_; *Q*_3_)	33 [0; 67]	33 [0; 33]	0 [0; 33]	
*Min*–*Max*	0–100	0–67	0–100	
Diarrhoea (DI)				**<0.001**
*M* ± *SD*	35.4 ± 33.8	6.3 ± 15.4	6.3 ± 17.5	
*Me* (*Q*_1_; *Q*_3_)	33 [0; 67]	0 [0; 0]	0 [0; 0]	
*Min*–*Max*	0–100	0–67	0–100	
Financial difficulties (FI)				**<0.001**
*M* ± *SD*	45.8 ± 32.5	33.3 ± 28.0	22.7 ± 28.4	
*Me* (*Q*_1_; *Q*_3_)	33 [33; 67]	33 [0; 33]	0 [0; 33]	
*Min*–*Max*	0–100	0–100	0–100	

*M*—mean; *SD*—standard deviation; *Me*—median; *Q*_1_—lower quartile; *Q*_3_—upper quartile; *Min*—lowest value; *Max*—highest value; *n*—number; values in bold indicate statistical significance (*p* < 0.05).

**Table 5:** The results of QoL assessment (EORTC QLQ-BR23) in patient groups differing with respect to the level of illness acceptance; and the results of the analysis of variance.

Functional Scales QLQ-BR23	Level of Illness Acceptance (AIS)			ANOVA *p*
	Low Acceptance 8–18 pts.	Moderate Acceptance 19–29 pts.	High Acceptance 30–40 pts.	
	*n* = 32	*n* = 69	*n* = 101	
Body image (BRBI)				**<0.001**
*M* ± *SD*	30.3 ± 25.9	46.3 ± 28.4	51.0 ± 25.2	
*Me* (*Q*_1_; *Q*_3_)	25 [8; 33]	42 [25; 67]	54 [33; 75]	
*Min*–*Max*	0–100	0–100	0–92	
Sexual functioning (BRSEF)				**0.001**
*M* ± *SD*	29.2 ± 29.9	43.5 ± 29.6	47.4 ± 29.1	
*Me* (*Q*_1_; *Q*_3_)	33 [0; 33]	33 [33; 67]	50 [33; 67]	
*Min*–*Max*	0–100	0–100	0–100	
Sexual enjoyment (BRSEE)				**<0.001**
*M* ± *SD*	14.7 ± 27.4	40.2 ± 31.7	46.1 ± 37.5	
*Me* (*Q*_1_; *Q*_3_)	0 [0; 33]	33 [0; 67]	33 [0; 67]	
*Min*–*Max*	0–100	0–100	0–100	
Future perspective (BRFU)				**0.010**
*M* ± *SD*	70.8 ± 29.0	80.9 ± 26.6	66.7 ± 32.0	
*Me* (*Q*_1_; *Q*_3_)	67 [33; 100]	100 [67; 100]	67 [33; 100]	
*Min*–*Max*	33–100	0–100	0–100	
QLQ-BR23 symptom scales				
Systemic therapy side effects (BRST)				**<0.001**
*M* ± *SD*	41.8 ± 20.9	32.9 ± 19.7	22.9 ± 17.1	
*Me* (*Q*_1_; *Q*_3_)	43 [28; 58]	29 [14; 43]	19 [10; 33]	
*Min*–*Max*	5–76	0–100	0–71	
Breast symptoms (BRBS)				**<0.001**
*M* ± *SD*	42.5 ± 30.2	33.7 ± 24.2	19.0 ± 20.8	
*Me* (*Q*_1_; *Q*_3_)	46 [8; 67]	33 [8; 50]	8 [0; 33]	
*Min*–*Max*	0–92	0–92	0–92	
Arm symptoms (BRAS)				**0.011**
*M* ± *SD*	45.1 ± 23.5	37.4 ± 26.1	31.0 ± 22.5	
*Me* (*Q*_1_; *Q*_3_)	44 [22; 58]	33 [11; 56]	22 [11; 44]	
*Min*–*Max*	0–89	0–89	0–100	
Upset by hair loss (BRHL)				0.141
*M* ± *SD*	54.7 ± 30.3	61.7 ± 35.9	46.8 ± 35.9	
*Me* (*Q*_1_; *Q*_3_)	67 [33; 67]	67 [33; 100]	33 [33; 67]	
*Min*–*Max*	0–100	0–100	0–100	

*M*—mean; *SD*—standard deviation; *Me*—median; *Q*_1_—lower quartile; *Q*_3_—upper quartile; *Min*—lowest value; *Max*—highest value; *n*—number; values in bold indicate statistical significance (*p* < 0.05).

**Table 6:** The results of QoL assessment (EORTC QLQ-C30 functional scales) in patient groups differing with respect to life orientation and the results of the analysis of variance.

QoL Assessed with EORTC QLQ-C30	Life Orientation (LOT-R)			ANOVA *p*
	Pessimistic Disposition 0–4 Sten Scores	Moderate Optimism 5–6 Sten Scores	Optimistic Disposition 7–10 Sten Scores	
	*n* = 33	*n* = 71	*n* = 97	
Global health status/QoL (QL)				**<0.001**
*M* ± *SD*	47.7 ± 22.4	54.5 ± 22.4	64.4 ± 16.5	
*Me* (*Q*_1_; *Q*_3_)	50 [33; 58]	58 [42; 67]	67 [50; 83]	
*Min*–*Max*	8–100	0–100	17–100	
Physical functioning (PF)				**0.003**
*M* ± *SD*	19.7 ± 13.3	28.1 ± 20.3	27.3 ± 18.3	
*Me* (*Q*_1_; *Q*_3_)	27 [13; 27]	20 [17; 37]	20 [13; 47]	
*Min*–*Max*	0–60	0–87	7–60	
Role functioning (RF)				**0.009**
*M* ± *SD*	15.1 ± 16.7	24.6 ± 25.8	23.7 ± 21.7	
*Me* (*Q*_1_; *Q*_3_)	17 [0; 17]	17 [0; 33]	17 [0; 50]	
*Min*–*Max*	0–67	0–100	0–67	
Emotional functioning (EF)				**<0.001**
*M* ± *SD*	29.7 ± 20.2	43.7 ± 27.1	47.2 ± 22.8	
*Me* (*Q*_1_; *Q*_3_)	25 [17; 33]	33 [25; 75]	42 [33; 67]	
*Min*–*Max*	0–92	0–100	0–92	
Cognitive functioning (CF)				**0.017**
*M* ± *SD*	19.6 ± 19.2	29.3 ± 29.5	30.3 ± 27.5	
*Me* (*Q*_1_; *Q*_3_)	17 [0; 33]	17 [0; 42]	33 [0; 50]	
*Min*–*Max*	0–83	0–100	0–83	
Social functioning (SF)				**0.002**
*M* ± *SD*	23.2 ± 20.5	35.0 ± 26.3	34.8 ± 26.1	
*Me* (*Q*_1_; *Q*_3_)	17 [0; 33]	33 [17; 50]	33 [17; 50]	
*Min*–*Max*	0–100	0–100	0–100	

*M*—mean; *SD*—standard deviation; *Me*—median; *Q*_1_—lower quartile; *Q*_3_—upper quartile; *Min*—lowest value; *Max*—highest value; *n*—number; %—percentage; values in bold indicate statistical significance (*p* < 0.05).

**Table 7:** The results of QoL assessment (EORTC QLQ-C30 symptom scales) in patient groups differing with respect to life orientation and the results of the analysis of variance.

QLQ-C30 Symptom Scales	Life Orientation (LOT-R)			ANOVA *p*
	Pessimistic Disposition 0–4 Sten Scores	Moderate Optimism 5–6 Sten Scores	Optimistic Disposition 7–10 Sten Scores	
	*n* = 33	*n* = 71	*n* = 97	
Fatigue (FA)				0.178
*M* ± *SD*	44.8 ± 23.2	40.8 ± 21.6	37.0 ± 21.5	
*Me* (*Q*_1_; *Q*_3_)	44 [33; 56]	33 [22; 56]	33 [22; 44]	
*Min*–*Max*	0–100	0–100	0–100	
Nausea and vomiting (NV)				**0.001**
*M* ± *SD*	25.8 ± 32.6	18.8 ± 27.6	7.7 ± 19.7	
*Me* (*Q*_1_; *Q*_3_)	0 [0; 50]	0 [0; 33]	0 [0; 0]	
*Min*–*Max*	0–100	0–100	0–100	
Pain (PA)				**0.006**
*M* ± *SD*	36.4 ± 24.1	31.9 ± 28.7	22.5 ± 21.4	
*Me* (*Q*_1_*; Q_3_*)	33 [17; 50]	33 [17; 50]	17 [0; 33]	
*Min*–*Max*	0–67	0–100	0–83	
Dyspnoea (DY)				**0.024**
*M* ± *SD*	18.2 ± 27.8	19.5 ± 30.3	9.3 ± 19.7	
*Me* (*Q*_1_; *Q*_3_)	0 [0; 33]	0 [0; 33]	0 [0; 0]	
Min–Max	0–67	0–100	0–100	
Sleep disturbance (SL)				0.325
*M* ± *SD*	36.5 ± 29.8	45.1 ± 28.2	38.1 ± 37.9	
*Me* (*Q*_1_; *Q*_3_)	33 [0; 67]	33 [33; 67]	33 [0; 67]	
*Min*–*Max*	0–100	0–100	0–100	
Appetite loss (AP)				**<0.001**
*M* ± *SD*	27.3 ± 31.7	29.1 ± 29.8	12.0 ± 22.1	
*Me* (*Q*_1_; *Q*_3_)	33 [0; 33]	33 [0; 67]	0 [0; 33]	
*Min*–*Max*	0–100	0–100	0–100	
Constipation (CO)				**0.003**
*M* ± *SD*	28.3 ± 26.5	25.8 ± 25.9	14.8 ± 22.0	
*Me* (*Q*_1_; *Q*_3_)	33 [0; 33]	33 [0; 33]	0 [0; 33]	
*Min*–*Max*	0–100	0–100	0–100	
Diarrhoea (DI)				**0.014**
*M* ± *SD*	18.2 ± 32.4	12.7 ± 22.1	6.2 ± 16.2	
*Me* (*Q*_1_; *Q*_3_)	0 [0; 33]	0 [0; 33]	0 [0; 0]	
*Min*–*Max*	0–100	0–67	0–100	
Financial difficulties (FI)				**0.007**
*M* ± *SD*	35.4 ± 33.3	37.1 ± 32.6	23.3 ± 25.2	
*Me* (*Q*_1_; *Q*_3_)	33 [0; 33]	33 [0; 67]	33 [0; 33]	
*Min*–*Max*	0–100	0–100	0–100	

*M*—mean; *SD*—standard deviation; *Me*—median; *Q*_1_—lower quartile; *Q*_3_—upper quartile; *Min*—lowest value; *Max*—highest value; *n*—number; values in bold indicate statistical significance (*p* < 0.05).

**Table 8:** The results of QoL assessment (EORTC QLQ-BR23) in patient groups differing with respect to the sense of coherence and the results of the analysis of variance.

Functional Scales QLQ-BR23	Life Orientation (LOT-R)			ANOVA *p*
	Pessimistic Disposition 0–4 Sten Scores	Moderate Optimism 5–6 Sten Scores	Optimistic Disposition 7–10 Sten Scores	
	*n* = 33	*n* = 71	*n* = 97	
Body image (BRBI)				**0.050**
*M* ± *SD*	34.1 ± 28.9	43.5 ± 26.8	44.2 ± 26.6	
*Me* (*Q*_1_; *Q*_3_)	33 [17; 50]	33 [25; 67]	50 [17; 67]	
*Min*–*Max*	0–100	0–100	0–92	
Sexual functioning (BRSEF)				0.142
*M* ± *SD*	39.4 ± 29.4	41.5 ± 29.1	32.5 ± 31.6	
*Me* (*Q*_1_; *Q*_3_)	33 [17; 67]	33 [17; 67]	33 [0; 50]	
*Min*–*Max*	0–100	0–100	0–100	
Sexual enjoyment (BRSEE)				**0.028**
*M* ± *SD*	27.3 ± 30.2	33.9 ± 35.0	46.3 ± 36.2	
*Me* (*Q*_1_; *Q*_3_)	33 [0; 33]	33 [0; 67]	33 [0; 67]	
*Min*–*Max*	0–100	0–100	0–100	
Future perspective (BRFU)				**0.049**
*M* ± *SD*	67.7 ± 31.9	74.6 ± 28.4	81.8 ± 25.1	
*Me* (*Q*_1_; *Q*_3_)	67 [33; 100]	67 [67; 100]	100 [67; 100]	
*Min*–*Max*	0–100	0–100	33–100	
Systemic therapy side effects (BRST)				**0.013**
*M* ± *SD*	33.1 ± 20.3	33.1 ± 22.2	24.9 ± 17.0	
*Me* (*Q*_1_; *Q*_3_)	33 [14; 52]	29 [14; 48]	24 [10; 33]	
*Min*–*Max*	0–71	0–100	0–71	
Breast symptoms (BRBS)				0.251
*M* ± *SD*	31.0 ± 26.5	30.3 ± 26.1	24.6 ± 24.2	
*Me* (*Q*_1_; *Q*_3_)	33 [8; 50]	33 [8; 50]	25 [0; 33]	
*Min*–*Max*	0–83	0–92	0–92	
Arm symptoms (BRAS)				0.487
*M* ± *SD*	36.0 ± 22.2	37.7 ± 26.0	33.2 ± 23.7	
*Me* (*Q*_1_; *Q*_3_)	33 [22; 56]	33 [22; 56]	33 [11; 44]	
*Min*–*Max*	0–78	0–89	0–100	
Upset by hair loss (BRHL)				0.415
*M* ± *SD*	61.9 ± 32.1	54.4 ± 34.0	49.6 ± 37.4	
*Me* (*Q*_1_; *Q*_3_)	67 [33; 100]	67 [33; 67]	33 [33; 100]	
*Min*–*Max*	0–100	0–100	0–100	

*M*—mean; *SD*—standard deviation; *Me*—median; *Q*_1_—lower quartile; *Q*_3_—upper quartile; *Min*—lowest value; *Max*—highest value; *n*—number; values in bold indicate statistical significance (*p* < 0.05).

**Table 9:** The results of QoL assessment (EORTC QLQ-C30) in patient groups differing with respect to illness perception and the results of the analysis of variance.

QoL Assessed with EORTC QLQ-C30	Illness Perception (MEDIS)			ANOVA *p*
	Positive 0–68 pts.	Neutral 69–94 pts.	Negative 95–140 pts.	
	*n* = 69	*n* = 68	*n* = 65	
Global health status/QoL (QL)				**<0.001**
*M* ± *SD*	65.1 ± 15.6	59.3 ± 19.8	49.2 ± 23.3	
*Me* (*Q*_1_; *Q*_3_)	67 [58; 75]	67 [50; 69]	50 [33; 67]	
*Min*–*Max*	25–100	8–100	0–100	
Physical functioning (PF)				**<0.001**
*M* ± *SD*	32.4 ± 21.4	22.3 ± 13.8	18.0 ± 13.0	
*Me* (*Q*_1_; *Q*_3_)	27 [17; 47]	20 [13; 27]	13 [7; 27]	
*Min*–*Max*	0–87	0–60	0–53	
Role functioning (RF)				**<0.001**
*M* ± *SD*	29.7 ± 25.9	17.9 ± 18.3	12.6 ± 15.8	
*Me* (*Q*_1_; *Q*_3_)	17 [17; 50]	17 [0; 33]	0 [0; 17]	
*Min*–*Max*	0–100	0–67	0–67	
Emotional functioning (EF)				**<0.001**
*M* ± *SD*	50.6 ± 25.8	33.0 ± 21.1	30.0 ± 21.5	
*Me* (*Q*_1_; *Q*_3_)	58 [33; 75]	33 [17; 42]	25 [17; 33]	
*Min*–*Max*	0–92	0–92	0–100	
Cognitive functioning (CF)				**<0.001**
*M* ± *SD*	34.6 ± 27.7	22.1 ± 21.2	18.1 ± 23.4	
*Me* (*Q*_1_; *Q*_3_)	33 [17; 50]	17 [0; 33]	17 [0; 33]	
*Min*–*Max*	0–100	0–83	0–100	
Social functioning (SF)				**<0.001**
*M* ± *SD*	46.7 ± 25.5	25.0 ± 19.8	17.1 ± 16.4	
*Me* (*Q*_1_; *Q*_3_)	50 [33; 67]	25 [17; 33]	17 [0; 33]	
*Min*–*Max*	0–100	0–67	0–67	

*M*—mean; *SD*—standard deviation; *Me*—median; *Q*_1_—lower quartile; *Q*_3_—upper quartile; *Min*—lowest value; *Max*—highest value; *n*—number; values in bold indicate statistical significance (*p* < 0.05).

**Table 10:** The results of QoL assessment (EORTC QLQ-C30 symptom scales) in patient groups differing with respect to illness perception and the results of the analysis of variance.

QLQ-C30 Symptom Scales	Illness Perception (MEDIS)			ANOVA *p*
	Positive 0–68 pts.	Neutral 69–94 pts.	Negative 95–140 pts.	
	*n* = 69	*n* = 68	*n* = 65	
Fatigue (FA)				**0.003**
*M* ± *SD*	33.2 ± 22.5	40.8 ± 22.0	45.8 ± 19.7	
*Me* (*Q*_1_; *Q*_3_)	33 [22; 33]	33 [31; 56]	44 [33; 56]	
*Min*–*Max*	0–100	0–100	11–89	
Nausea and vomiting (NV)				**<0.001**
*M* ± *SD*	8.2 ± 19.9	7.4 ± 17.1	29.5 ± 32.6	
*Me* (*Q*_1_; *Q*_3_)	0 [0; 0]	0 [0; 0]	17 [0; 50]	
*Min*–*Max*	0–100	0–100	0–100	
Pain (PA)				**<0.001**
*M* ± *SD*	21.7 ± 25.0	24.5 ± 21.3	39.2 ± 26.1	
*Me* (*Q*_1_; *Q*_3_)	17 [0; 33]	17 [0; 33]	33 [17; 67]	
*Min*–*Max*	0–100	0–67	0–100	
Dyspnoea (DY)				**0.028**
*M* ± *SD*	12.1 ± 22.1	10.3 ± 21.7	21.4 ± 31.1	
*Me* (*Q*_1_; *Q*_3_)	0 [0; 33]	0 [0; 0]	0 [0; 33]	
*Min*–*Max*	0–100	0–100	0–100	
Sleep disturbance (SL)				**0.045**
*M* ± *SD*	38.2 ± 35.4	34.8 ± 35.0	48.7 ± 28.3	
*Me* (*Q*_1_; *Q*_3_)	33 [0; 67]	33 [0; 67]	33 [33; 67]	
*Min*–*Max*	0–100	0–100	0–100	
Appetite loss (AP)				**<0.001**
*M* ± *SD*	14.5 ± 24.6	14.7 ± 21.1	33.8 ± 33.1	
*Me* (*Q*_1_; *Q*_3_)	0 [0; 33]	0 [0; 33]	33 [0; 67]	
*Min*–*Max*	0–100	0–67	0–100	
Constipation (CO)				**0.002**
*M* ± *SD*	15.0 ± 22.5	18.6 ± 24.7	29.2 ± 25.3	
*Me* (*Q*_1_; *Q*_3_)	0 [0; 33]	0 [0; 33]	33 [0; 33]	
*Min*–*Max*	0–100	0–100	0–100	
Diarrhoea (DI)				**0.002**
*M* ± *SD*	5.8 ± 16.1	8.3 ± 21.8	19.0 ± 27.6	
*Me* (*Q*_1_; *Q*_3_)	0 [0; 0]	0 [0; 0]	0 [0; 33]	
*Min*–*Max*	0–100	0–100	0–100	
Financial difficulties (FI)				**<0.001**
*M* ± *SD*	20.6 ± 28.2	24.0 ± 25.0	46.2 ± 30.4	
*Me* (*Q*_1_; *Q*_3_)	0 [0; 33]	33 [0; 33]	33 [33; 67]	
*Min*–*Max*	0–100	0–100	0–100	

*M*—mean; *SD*—standard deviation; *Me*—median; *Q*_1_—lower quartile; *Q*_3_—upper quartile; *Min*—lowest value; *Max*—highest value; *n*—number; values in bold indicate statistical significance (*p* < 0.05).

**Table 11:** The results of QoL assessment (EORTC QLQ-BR23) in patient groups differing with respect to illness perception and the results of the analysis of variance.

Functional Scales QLQ-BR23	Illness Perception (MEDIS)			ANOVA *p*
	Positive 0–68 pts.	Neutral 69–94 pts.	Negative 95–140 pts.	
	*n* = 69	*n* = 68	*n* = 65	
Body image (BRBI)				**0.001**
*M* ± *SD*	56.1 ± 26.8	33.6 ± 26.2	28.3 ± 23.3	
*Me* (*Q*_1_; *Q*_3_)	58 [33; 75]	33 [17; 50]	33 [8; 33]	
*Min*–*Max*	0–100	0–100	0–92	
Sexual functioning (BRSEF)				**<0.001**
*M* ± *SD*	53.8 ± 27.3	32.8 ± 31.3	25.1 ± 25.7	
*Me* (*Q*_1_; *Q*_3_)	50 [33; 67]	33 [0; 50]	33 [0; 33]	
*Min*–*Max*	0–100	0–100	0–100	
Sexual enjoyment (BRSEE)				0.157
*M* ± *SD*	43.5 ± 36.5	42.3 ± 36.2	31.4 ± 33.3	
*Me* (*Q*_1_; *Q*_3_)	33 [0; 67]	33 [0; 67]	33 [0; 67]	
*Min*–*Max*	0–100	0–100	0–100	
Future perspective (BRFU)				**<0.001**
*M* ± *SD*	80.0 ± 26.9	76.5 ± 29.4	60.3 ± 31.2	
*Me* (*Q*_1_; *Q*_3_)	100 [67; 100]	100 [33; 100]	67 [33; 100]	
*Min*–*Max*	0–100	33–100	0–100	
Systemic therapy side effects (BRST)				**<0.001**
*M* ± *SD*	21.9 ± 15.9	28.5 ± 18.9	37.9 ± 21.4	
*Me* (*Q*_1_; *Q*_3_)	19 [10; 29]	29 [14; 43]	33 [24; 57]	
*Min*–*Max*	0–67	0–71	0–100	
Breast symptoms (BRBS)				**<0.001**
*M* ± *SD*	17.2 ± 20.9	26.6 ± 25.3	40.0 ± 24.5	
*Me* (*Q*_1_; *Q*_3_)	8 [0; 29]	21 [8; 33]	42 [25; 58]	
*Min*–*Max*	0–92	0–92	0–92	
Arm symptoms (BRAS)				**<0.001**
*M* ± *SD*	24.6 ± 21.7	36.3 ± 22.4	46.0 ± 24.4	
*Me* (*Q*_1_; *Q*_3_)	22 [11; 33]	33 [22; 56]	44 [22; 67]	
*Min*–*Max*	0–100	0–89	0–89	
Upset by hair loss (BRHL)				**<0.001**
*M* ± *SD*	37.1 ± 36.9	50.5 ± 34.3	68.9 ± 27.6	
*Me* (*Q*_1_; *Q*_3_)	33 [0; 67]	33 [33; 67]	67 [67; 100]	
*Min*–*Max*	0–100	0–100	0–100	

*M*—mean; *SD*—standard deviation; *Me*—median; *Q*_1_—lower quartile; *Q*_3_—upper quartile; *Min*—lowest value; *Max*—highest value; *n*—number; values in bold indicate statistical significance (*p* < 0.05).

**Table 12:** Pearson’s correlation coefficient values (*r*) for QoL (EORTC QLQ-C30 and BR23) and illness perception (MEDIS).

QLQ-C30 and BR-23	MEDIS				
	SC	MD	PD	IN	SW
QL	**−0.362**	**−0.276**	**−0.184**	**−0.215**	**−0.309**
PF	**−0.394**	**−0.279**	**−0.212**	**−0.203**	**−0.327**
RF	**−0.378**	**−0.267**	**−0.217**	**−0.234**	**−0.347**
EF	**−0.363**	**−0.353**	−0.124	**−0.192**	**−0.303**
CF	**−0.316**	**−0.212**	−0.073	**−0.184**	**−0.298**
SF	**−0.500**	**−0.426**	**−0.289**	**−0.221**	**−0.430**
FA	**0.285**	**0.178**	**0.287**	**0.254**	**0.191**
NV	**0.402**	**0.280**	**0.149**	**0.168**	**0.343**
PA	**0.323**	**0.227**	**0.179**	**0.144**	**0.243**
DY	**0.201**	**0.173**	0.069	0.102	**0.271**
SL	0.134	**0.178**	**0.170**	0.065	**0.140**
AP	**0.329**	**0.220**	0.119	0.156	**0.252**
CO	**0.268**	**0.225**	**0.177**	0.070	**0.300**
DI	**0.296**	**0.213**	**0.177**	**0.184**	**0.270**
FI	**0.378**	**0.314**	0.135	0.138	**0.377**
BRBI	**−0.383**	**−0.387**	**−0.174**	−0.104	**−0.384**
BRSEF	**−0.402**	**−0.346**	**−0.189**	−0.083	**−0.366**
BRSEE	**−0.207**	−0.078	−0.067	0.075	**−0.178**
BRFU	**−0.224**	**−0.270**	**−0.173**	**−0.260**	**−0.145**
BRST	**0.377**	**0.270**	**0.167**	**0.205**	**0.283**
BRBS	**0.367**	**0.329**	**0.195**	**0.213**	**0.374**
BRAS	**0.374**	**0.388**	**0.236**	**0.164**	**0.316**
BRHL	**0.362**	**0.276**	**0.184**	**0.215**	**0.309**

Illness perception: SC—self-realization constraints; MD—mental dysfunction; PD—physical dysfunction; IN—infection; SW—social withdrawal; linear correlation coefficients other than zero at *p* < 0.05 are marked in bold.

**Table 13:** The results of life optimism assessment (LOT-R) in patient groups differing with respect to the degree of using constructive strategies of coping with cancer and the results of the analysis of variance.

Life Orientation (LOT-R)	Constructive Strategies of Coping with Cancer (Mini-MAC)						ANOVA *p*
	Low 0–4 Sten Scores		Average 5–6 Sten Scores		High 7–10 Sten Scores		
	*n* = 5		*n* = 69		*n* = 127		
Level of dispositional optimism (sten scores):							**0.010**
*M* ± *SD*	4.0 ± 3.5		6.2 ± 1.7		6.5 ± 1.9		
*Me* (*Q*_1_; *Q*_3_)	3 [2; 4]		6 [5; 7]		7 [5; 8]		
*Min*–*Max*	1–10		2–10		2–10		
LOT-R results:	*n*	%	*n*	%	*n*	%	**0.002**
Pessimistic disposition	4	80.0%	13	18.8%	16	12.6%	
Moderate optimism	0	0.0%	25	36.2%	46	36.2%	
Optimistic disposition	1	20.0%	31	44.9%	65	51.2%	
Level of illness acceptance (AIS; pts.)							**0.006**
*M* ± *SD*	16.6 ± 4.0		29.0 ± 8.6		28.8 ± 8.3		
*Me* (*Q*_1_; *Q*_3_)	17 [17; 18]		31 [24; 36]		29 [24; 36]		
*Min*–*Max*	10–21		8–40		8–40		

*M*—mean; *SD*—standard deviation; *Me*—median; *Q*_1_—lower quartile; *Q*_3_—upper quartile; *Min*—lowest value; *Max*—highest value; *n*—number; %—percentage; values in bold indicate statistical significance (*p* < 0.05).

**Table 14:** The results of life optimism assessment (LOT-R) in patient groups differing with respect to the degree of using destructive strategies of coping with cancer and the results of the analysis of variance.

Life Orientation (LOT-R)	Destructive Strategies of Coping with Cancer (Mini-MAC)						ANOVA *p*
	Low 0–4 Sten Scores		Average 5–6 Sten Scores		High 7–10 Sten Scores		
	*n* = 125		*n* = 59		*n* = 17		
Level of dispositional optimism (sten scores):							**<0.001**
*M* ± *SD*	6.9 ± 1.8		5.7 ± 1.8		4.5 ± 1.2		
*Me* (*Q*_1_; *Q*_3_)	7 [6; 8]		6 [5; 7]		5 [4; 5]		
*Min*–*Max*	2–10		2–9		1–6		
LOT-R results:							**<0.001**
Pessimistic disposition	12	9.6%	15	25.4%	6	35.3%	
Moderate optimism	37	29.6%	23	39.0%	11	64.7%	
Optimistic disposition	76	60.8%	21	35.6%	0	0.0%	
Level of illness acceptance (AIS; pts.)							**<0.001**
*M* ± *SD*	30.9 ± 7.6		27.3 ± 7.0		15.1 ± 6.0		
*Me* (*Q*_1_; *Q*_3_)	33 [27; 37]		26 [24; 32]		13 [11; 18]		
*Min*–*Max*	8–40		12–40		8–29		

*M*—mean; *SD*—standard deviation; *Me*—median; *Q*_1_—lower quartile; *Q*_3_—upper quartile; *Min*—lowest value; *Max*—highest value; *n*—number; %—percentage; values in bold indicate statistical significance (*p* < 0.05).

## Data Availability

All the data of this study are available from the corresponding author on reasonable request.
